# Is there hope that transpinal direct current stimulation corrects motoneuron excitability and provides neuroprotection in amyotrophic lateral sclerosis?

**DOI:** 10.14814/phy2.14706

**Published:** 2021-01-19

**Authors:** Marcin Bączyk, Piotr Krutki, Daniel Zytnicki

**Affiliations:** ^1^ Department of Neurobiology Poznan University of Physical Education Poznań Poland; ^2^ Université de Paris, Centre National de la Recherche Scientifique (CNRS) Saints‐Pères Paris Institute for the Neurosciences (SPPIN) Paris France

**Keywords:** excitability and excitation, intracellular recordings, neurodegenerative disease, spinal motoneurons

## Abstract

Amyotrophic lateral sclerosis (ALS) is a fatal neurodegenerative disease of largely unknown pathophysiology, characterized by the progressive loss of motoneurons (MNs). We review data showing that in presymptomatic ALS mice, MNs display reduced intrinsic excitability and impaired level of excitatory inputs. The loss of repetitive firing specifically affects the large MNs innervating fast contracting muscle fibers, which are the most vulnerable MNs in ALS. Interventions that aimed at restoring either the intrinsic excitability or the synaptic excitation result in a decrease of disease markers in MNs and delayed neuromuscular junction denervation. We then focus on trans‐spinal direct current stimulation (tsDCS), a noninvasive tool, since it modulates the activity of spinal neurons and networks. Effects of tsDCS depend on the polarity of applied current. Recent work shows that anodal tsDCS induces long‐lasting enhancement of MN excitability and synaptic excitation of spinal MNs. Moreover, we show preliminary results indicating that anodal tsDCS enhances the excitatory synaptic inputs to MNs in ALS mice. In conclusion, we suggest that chronic application of anodal tsDCS might be useful as a complementary method in the management of ALS patients.

## Graphical abstract

Motoneuron activity is disrupted during Amyotrophic lateral sclerosis (ALS), depriving these cells of activity‐dependent neuroprotection. Anodal tsDCS can modify motoneuron excitability promising a new approach for ALS management.

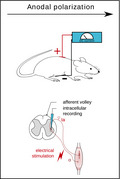

## 1 INTRODUCTION

Recent data demonstrate that the excitability changes of spinal motoneurons (MNs) in amyotrophic lateral sclerosis (ALS, a prominent neurodegenerative disease of MNs) depend on the physiological type of motor unit and evolve with disease progression. Most interestingly, interventions with pharmacological or chemogenetic tools that aim at correcting the firing of the most vulnerable MNs prove to have some beneficial impact on the disease. After reviewing these data, we focus on trans‐spinal direct current stimulation (tsDCS) as a potential alternative therapeutic method in ALS. Indeed, electrical polarization by direct current is well‐known to modify spinal networks. We review recent work suggesting that tsDCS could be used to compensate for the changes in intrinsic excitability of MNs, or synaptic excitation, and hopefully to deliver some neuroprotection in ALS.

### 1.1 Changes of electrical properties of MNs in ALS

In ALS, some motor pools are more vulnerable than others (Kanning et al., 2010), but even within a given motor pool, the order of MN degeneration depends on MN type: fast contracting—fatigable (FF) motor units degenerate first, followed by fast contracting—fatigue‐resistant units (FR), whereas slow contracting motor units (S) are resistant to degeneration (Hegedus et al., 2008; Pun et al., 2006). Despite more than 20 years of intense research, the pathophysiological mechanisms that lead to MN degeneration in ALS are still largely unknown. Among many others, the glutamate excitotoxic hypothesis has been proposed (Ilieva et al., 2009; Van Den Bosch et al., 2006), which relies on the assumption that excessive excitatory glutamatergic input may lead to an overload of cytosolic calcium, through calcium permeable‐α‐amino‐3‐hydroxy‐5‐methyl‐4‐isoxazolepropionic acid (AMPA) and N‐methyl‐D‐aspartatic acid (NMDA) channels and voltage‐dependent calcium channels activated by action potentials, which, in turn, triggers apoptosis.

One argument that supports the glutamate excitotoxic hypothesis is the fact that Riluzole (which was for a long time the only FDA‐approved treatment for ALS) prolongs survival in patients with ALS, albeit modestly (a few months, Bensimon et al., 1994; Miller et al., 2003). Indeed, Riluzole has multiple actions, among those a decrease of glutamatergic transmission and intrinsic excitability—notably by blocking the persistent inward sodium current (Bellingham, 2013; Kuo et al., 2006; Lamanauskas & Nistri, 2008). However, it was recently shown that the survival benefit of Riluzole is achieved by extending the fourth stage of the disease, in which the motor functions are largely impaired and which immediately precedes death (Fang et al., 2018). Moreover, all other pharmacological interventions targeting the reduction of glutamate release or excitability have never worked in humans (Wobst et al., 2020). In particular, phase III clinical trials with Talampanel, an AMPA receptor antagonist, Memantine, an NMDA receptor antagonist, or Mexiletine, a sodium channel blocker, have not been conclusive (De Carvalho et al., 2010; Pascuzzi et al., 2010; Weiss et al., 2016). These results challenge the glutamatergic excitotoxicity hypothesis. However, intraspinal infusion of large doses of AMPA can induce excitotoxic MN degeneration in vivo, even in wild type animals (Netzahualcoyotzi & Tapia, 2015) but there is no evidence that such an intervention reproduces the action of synaptic glutamate release in ALS. Indeed, in ALS mouse models, AMPA receptor antagonists (Akamatsu et al., 2016; Van Damme et al., 2003) and NMDA receptor antagonists (Joo et al., 2007), delivered from symptom onset until death, elicit modest improvement of motor function and the survival time. This may not be so surprising, since the most vulnerable MNs have already degenerated at symptoms onset (Hegedus et al., 2008; Pun et al., 2006). Saxena et al. (2013) started the administration of the drugs as early as P20 in the SOD1 G93A mice and under these conditions, AMPA (a) ameliorated cellular markers of the disease in MNs (less misfolded SOD1 proteins, reduced unfolded protein response and stress of the endoplasmic reticulum), (b) delayed the denervation of the less vulnerable motor units, (c) improved the force of contraction, and (d) extended the survival by 20–35 days while AMPA antagonists had opposite effects. These results are in contrast with previous studies and contradict the glutamate excitotoxicity theory in ALS and suggest that the time of drug application, the cumulative dose, and the peak CNS concentration may somehow account for these conflicting results.

Indeed, in ALS mouse models, time‐dependent alterations of intrinsic MN excitability that start long before degeneration onset have been shown in both vulnerable and resistant MNs. In these studies it was found that MNs are hyperexcitable at embryonic stages in the SOD1 ^G93A^mice (input resistance is increased, rheobase is decreased and slope of the frequency–current relationship is increased; Martin et al., 2013; Pieri et al., 2003). When MNs were examined shortly after birth (P4–P10), contradictory results have been reported: in some works MN are hyperexcitable (van Zundert et al., 2008), in others they are hypoexcitable (Bories et al., 2007), or they do not display any change in excitability (Pambo‐Pambo et al., 2009) suggesting an efficient excitability homeostasis (Quinlan et al., 2011). However none of these studies have sorted MNs according to their physiological type, which might explain the reported discrepancies. More recently, Leroy et al. (2014) classified spinal MNs according to their discharge pattern, their anatomy and the expression of specific molecular markers within F‐ and S‐types in P6‐P10 mice and found that only S‐type MNs (the less vulnerable ones) are hyperexcitable (lower rheobase) at this age in the SOD1^G93A^ mice while F‐type MNs (the most vulnerable ones) display a normal excitability. A similar conclusion was reached by Venugopal et al. (2015) in trigeminal MNs.

**FIGURE 1:**
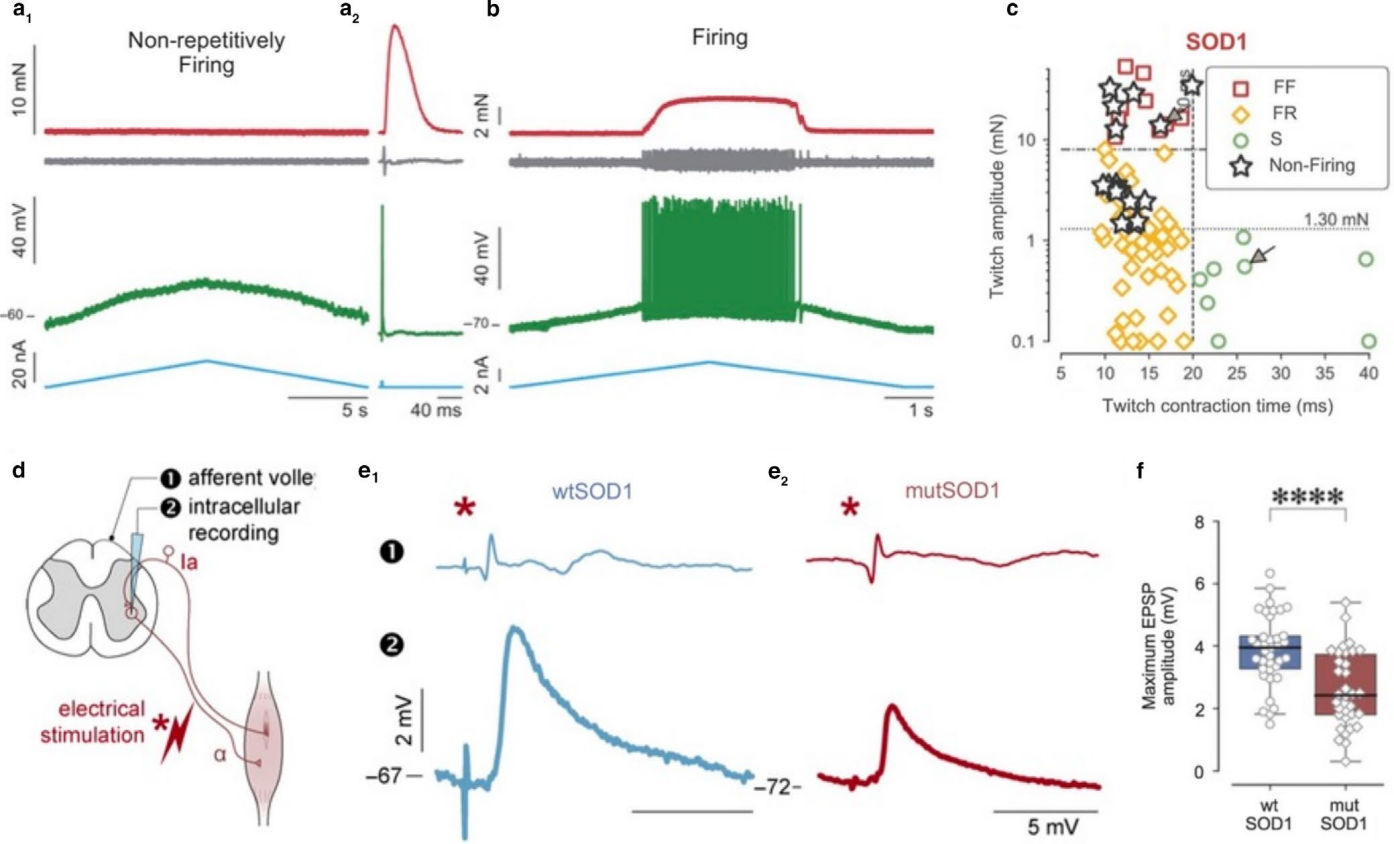
Intrinsic excitability and synaptic excitation are depressed in the SOD1^G93A^ mice. (a) MN that fails to display a repetitive discharge in response to a slow triangular ramp of current (A_1_) but that is still able to elicit a single spike in response to a short transient pulse (A_2_). The blue trace is the injected current, the green trace is the intracellular recording, the gray trace is the EMG recorded in the triceps surae, the red trace is the force recorded at the tendon of the triceps surae. (b) MN displaying a repetitive firing in response to a slow triangular ramp (same arrangement as in (a). (c) Nonfiring MNs are found among the largest motor units (FF and FR with a twitch force larger than 1.3 mN). MNs indicated by an arrow correspond to the two examples in (a) and (b). Adapted from Martinez‐Silva et al. (2018). (d) Experimental arrangement for testing the size of the maximal Ia Excitatory Post‐Synaptic Potentials (EPSP). (e) Typical recordings in a wtSOD1 MN (E_1_) and a SOD1^G93A^ MN (E_2_). Lower traces are the intracellular recordings. Upper traces are the cord dorsum potentials showing the group I afferent volleys. (f) The maximal Ia EPSPs are significantly reduced in the MNs from SOD1^G93A^ mice (whereas resting potentials, input conductances and membrane time constants are unchanged). Adapted from Bączyk, Alami et al. (2020)

However, the excitability pattern evolves during animal maturation. *In vivo* intracellular recordings in anesthetized mice allowed investigations in adults just prior to the degeneration of neuromuscular junctions of the most vulnerable motor units (P50–60 in SOD1^G93A^ mice). In these conditions, Delestrée et al. (2014) showed that a fraction of MNs lose their ability to fire repetitively in response to a slow ramp of current despite being functionally connected to their muscle fibers (Figure 1a). This loss of firing is interpreted as a manifestation of hypoexcitability. The ability to type‐identify motor units *in vivo* allowed Martínez‐Silva et al. (2018) to demonstrate that the loss of repetitive firing in SOD1^G93A^ mice (and also in an unrelated ALS model, FUS^P525L^; Sharma et al., 2016) occurred among the population of the most vulnerable MNs (innervating FF and largest FR motor units), while the most resistant MNs (smallest FR and S‐type motor units) display normal excitability (Figure 1c). Importantly, disease markers (p‐eIF2α and p62 aggregates) confirm that nonrepetitively firing MNs are in a more advanced stage of the disease than those that can still discharge normally (Martínez‐Silva et al., 2018). It should be noted that the Meehan group has not reported such hypoexcitability either in the SOD1^G127X^ mice (Meehan et al., 2010) or in the SOD1^G93A^ mice (Jensen et al., 2020). This discrepancy may be due to the fact that they used older mice, in which the most vulnerable MNs have already started to degenerate, than in the previous studies (Delestrée, 2014; Martínez‐Silva et al., 2018), as well as suboptimal discontinuous current‐clamp switching rate, which may distort the firing of those cells (Manuel, 2020).

Remarkably, recordings in induced pluripotent stem cell (iPSC)‐derived MNs from human patients have consistently reported the same time‐dependent excitability changes as in ALS mice. After an initial hyperexcitability (Devlin et al., 2015; Wainger et al., 2014) the cells turn into a hypoexcitable state as the cells mature (Devlin et al., 2015; Naujock et al., 2016; Sareen et al., 2013). A recent study found that the loss of repetitive discharge in IPSC‐derived MNs occurs only in presence of mutant astrocytes, indicating that these processes involve nonneuronal cell‐types (Zhao et al., 2020).

A major criticism that one may raise about in vivo pharmacological interventions is that such interventions act not only on MNs but also on all other neurons, including many classes of excitatory and inhibitory interneurons that provide inputs to MNs. Since MNs receive similar numbers of inhibitory and excitatory synapses (Bae et al., 1999), and since MNs were reported to be driven by balanced excitatory and inhibitory synaptic activity (Berg et al., 2007), the net effect of any drug on MN synaptic inputs is unpredictable and depends on the individual sensitivity of excitatory and inhibitory interneurons to the drug as well as to the synaptic composition and balance of each MN subpopulation.

Genetic interventions may also have an impact on both excitation and inhibition. For instance, Lalancette‐Hebert et al. (2016) demonstrated, in an ALS mouse model, that a genetic ablation of gamma MNs, which reduced the spindle activation, increases the mouse survival time. However, while Ia proprioceptive spindle afferents excite MNs they also very efficiently excite inhibitory interneurons which act on MNs; in particular interneurons which mediate the so‐called Ia reciprocal inhibition (Baldissera et al. 1981). The net effect on MNs (less excitation or less inhibition) of genetic ablation of gamma MNs is therefore unclear, and prevents a consistent conclusion for the mechanism responsible for the extended survival. To investigate the mechanism at work, interventions must selectively target alpha MNs, or excitatory or inhibitory synapses acting onto them.

**FIGURE 2:**
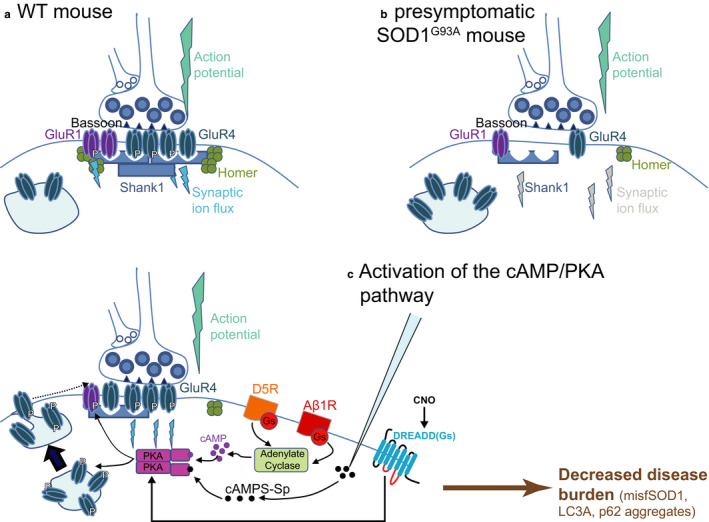
Excitatory synapses onto MNs are impaired in the SOD1^G93A^ mice and are restored through activation of the cAMP/PKA signaling pathway. (a) Drawing illustrating a normal excitatory synapse in a WT mouse. (b) In the presymptomatic SOD1^G93A^ mice (~ 50 days old), the GLUR subunits of the AMPA receptors and the scaffold proteins (Shank1, Homer) are less expressed in the postsynaptic side of excitatory synapses. At the same time, the presynaptic element does not seem affected. The postsynaptic disruption is responsible for a significant decrease of the EPSP amplitude (Bączyk, Alami et al., 2020). (c) Activation of the cAMP/PKA pathway, either through intracellular iontophoretic ejection of Sp‐AMP (a cAMP agonist) or through CNO‐activation of a DREADD G(s) specifically inserted in MN using an AAV9 vector, partially restores the synaptic impairment, entailing a firing increase and a burden decrease of disease markers such as misfSOD1, LC3A and p62 aggregates (Bączyk, Alami et al., 2020). The authors would like to thank Prof. Francesco Roselli who has drawn a preliminary draft of this figure and Dr. Marin Manuel who has prepared the final version

In this line, Saxena et al. (2013) performed *in vivo* chemogenetic manipulations, using viral vectors specifically targeting lumbar MNs in adult presymptomatic double transgenic SOD1^G93A^/ChAT‐cre mice. The virus expressed in MNs, the pharmacologically selective actuator module either coupled to 5HT3‐transmembrane domain for neuronal depolarization (to enhance the excitability), or to glycine‐receptor transmembrane domain for neuronal hyperpolarization (to decrease the excitability) (Magnus et al. 2011). Enhancing MN excitability reduced the amount of misfolded SOD1 protein, the endoplasmic reticulum stress in the FF‐type MNs, and delayed the denervation of the neuromuscular junctions in the corresponding MUs. Conversely, reducing MN excitability had opposite effects (Saxena et al., 2013). This seminal work demonstrated a causal link between changes in MN intrinsic excitability and vulnerability. Furthermore, recent experiments targeting excitatory synapses on MNs of ALS mice (Bączyk, Alami et al., 2020) showed that in presymptomatic SOD1^G93A^ mice, monosynaptic Excitatory Post‐Synaptic Potentials (EPSPs) (either from Ia spindle afferents or from descending systems) are functionally depressed in spinal MN (EPSPs are about 30% smaller, Figure 1d–f). This depression is caused by a molecular disruption of the postsynaptic cell (reduced amount of GluR subunits and scaffolded proteins, Figure 2; Bączyk, Alami et al., 2020). Bączyk, Alami et al. (2020) also demonstrated that the synaptic impairment can be rescued using a viral vector to express a DREADD(Gs) specifically into MNs of the double transgenic SOD1^G93A^/ChAT‐cre mice. Pharmacologic activation of DREADD(Gs) activated the cAMP/PKA signaling pathway eliciting membrane insertion of GluR4 subunits and restoration of excitatory synapses. This elicits an improvement of disease markers (misfolded SOD1 proteins, LC3A autophagic structures and P62 inclusions) through the enhancement of neuronal firing (Figure 2c; Bączyk, Alami et al., 2020). This work demonstrated that reduced excitation of MN at a presymptomatic stage contributes to the pathogenesis in ALS. On the contrary, increased excitation provides some neuroprotection of MNs.

Altogether, recent experiments, specifically targeting MNs, show that restoring MN intrinsic excitability or synaptic excitation has a beneficial impact on the disease pathobiochemistry. Gene therapy might then be envisioned to produce such a restoration. However, human gene therapy still requires a lot of development in order to (a) massively and specifically target MN, (b) control the cellular and immune responses, and (c) avoid side effects such as neoplastic tumors. There is evidently a real interest to develop noninvasive methods that could restore intrinsic excitability or synaptic excitation of MNs. In this framework, we will present new data showing that tsDCS may actually induce long‐lasting restoration of MN excitability and synaptic inputs, with the hope that tsDCS has the potential to deliver some neuroprotection in ALS.

### 1.2 Translational implications of direct current stimulation

The idea that electrical fields can influence the activity of spinal networks was introduced quite early. In the classical experiments it was already clear that the membrane of neurons is traversed by ionic currents that are responsible not only for action potentials (Hodgkin & Huxley, 1952), but also for excitatory and inhibitory postsynaptic potentials (Coombs et al., 1957). Spinal polarization was soon shown to modify the effectiveness of synaptic activation (Eccles et al., 1962) indicating that direct current stimulation can alter membrane ionic currents. Externally applied electrical currents gained further recognition as a neuromodulatory technique at the turn of the new century with the introduction of trans‐*cranial* direct current stimulation (tDCS) (Nitsche & Paulus, 2000). In this technique, direct current applied by electrodes located on the scalp modifies the activity of both cortical (Nitsche & Paulus, 2000) and subcortical (Bączyk & Jankowska, 2014; Bolzoni et al., 2013) regions. Therapeutic benefits of tDCS have recently been reported in motor rehabilitation (Bai et al., 2019), pain management (Ramger et al., 2019) and even psychiatric disorders (Kuo et al., 2017).

tsDCS was introduced by Cogiamanian et al. (2008) to induce long‐lasting alterations of the conduction velocity in the human lemniscal pathway. Excitability alteration of neuronal tracts by tsDCS was then shown to reduce nociception (Cogiamanian et al., 2011; Truini et al., 2011) or to modify H‐reflex (Lamy et al., 2012; Winkler et al., 2010). Subsequently tsDCS was used for a variety of treatments including to ameliorate idiopathic restless leg symptoms (Heide et al., 2014), modulate cortico‐spinal excitability (Bocci et al., 2015; Knikou et al., 2015; Murray et al., 2018), improve motor unit recruitment (Bocci et al., 2014), aid motor rehabilitation (Hubli et al., 2013), reduce spasticity (Ardolino et al., 2018; Paget‐Blanc et al., 2019), and reduce pain (Berra et al., 2019; Choi et al., 2019).

### 1.3 Effects of TSDCS on spinal neuronal networks

Despite a large number of translational studies, our basic knowledge of how tsDCS affects neuronal networks remains limited. It is already clear that tsDCS affects not only nerve fibers and spinal tract excitability, but it can also modulate intraspinal connectivity (Lenoir et al., 2018). Extensive animal studies have provided further evidence that tsDCS can directly affect spinal MN activation through both synaptic and axonal mechanisms (Ahmed, 2014). Significant improvement of our understanding of tsDCS actions came from the works of the E. Jankowska group who methodologically investigated how tsDCS influences the excitability of cutaneous and Ia afferents (Bolzoni & Jankowska, 2015), activity‐independent plasticity (Jankowska et al., 2016), myelinated nerve fibers activity (Jankowska, 2017), postactivation depression and presynaptic inhibition (Kaczmarek et al., 2017), as well as excitability of nerve fibers and their terminal branches in the presence of 4‐aminopyridine (Kaczmarek & Jankowska, 2018). The most recent study from the group suggests that branching points of primary afferent fibers are especially sensitive to DC current (Li et al., 2020).

One may wonder whether tsDCS mimics the actions of the spinal cord polarization produced by the electrical fields present around active neurons. Some electrical fields have amplitudes on the order of several millivolts, and can be detected at significant distances from cells active during locomotion (Noga et al., 1995). It is therefore possible that these fields influence the activity of neighboring cells and this concept was tested by Nelson (1966) in an elegant experiment that demonstrated MN activity can be influenced by subthreshold activation of synergistic neurons. More recently a similar concept was tested by Bączyk and Jankowska (2018) who showed that the excitability of myelinated nerve fibers can be modified by local field potentials evoked by stimulation of peripheral afferents.

The effects of tsDCS depend on the polarity of the applied current. In mice, spinal cord excitability was increased following cathodal polarization, but reduced during anodal tsDCS (Ahmed, 2014). In rats, the electromyographic responses from reticulospinal and rubrospinal pathways were facilitated by cathodal tDCS and depressed by anodal tDCS (Bolzoni et al., 2013). Furthermore, intraspinally applied cathodal current replicated the effects of tsDCS and strongly increased MN synaptic excitation by acting on the afferents to MNs, and these actions were consistently facilitatory with cathodal DC and depressive with anodal DC (Bolzoni & Jankowska, 2015; Kaczmarek & Jankowska, 2018). On the other hand, presynaptic inhibition and post activation depression were both facilitated by tsDCS in a polarity‐independent fashion (Kaczmarek et al., 2017). In the cat, in contrast to rats, anodal tDCS facilitated the activation of reticulospinal neurons (Bolzoni et al., 2013) and the actions of pyramidal tracts on MNs (Bączyk et al., 2014).

One important feature of tsDCS is its long‐term effects. Multiple studies performed both in human (Berry et al., 2017; Bolzoni et al., 2017; Kuck et al., 2018) and animal preparations (Bączyk & Jankowska, 2018; Bolzoni & Jankowska, 2015; Jankowska, 2017) indicate that the effects of polarization last up to several hours after the cessation of the stimulation. This phenomenon is of crucial importance when designing tsDCS interventions aimed at inducing long‐term neuromodulation.

### 1.4 Effects of TSDCS on intrinsic and synaptic properties of MNS

**FIGURE 3:**
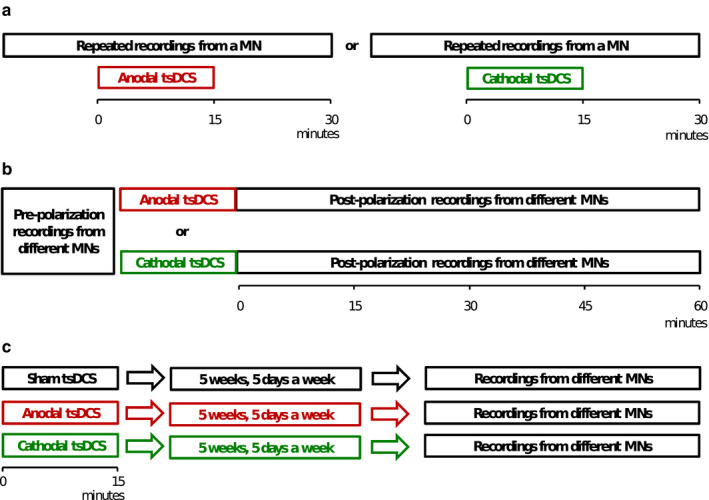
Schematic diagram of the experimental designs to investigate short‐term (a), persistent, long‐lasting (b) effects of acute tsDCS, and adaptive changes in response to chronic tsDCS application (c) in rat MNs

How does spinal polarization act at the level of a single spinal MN? The recent development of intracellular MN recordings coupled with spinal polarization *in vivo* (Bączyk & Krutki, 2020), enabled the direct investigation of short‐term, long‐lasting and chronic tsDCS‐induced alterations of MN electrophysiological properties (Figure 3).

**FIGURE 4:**
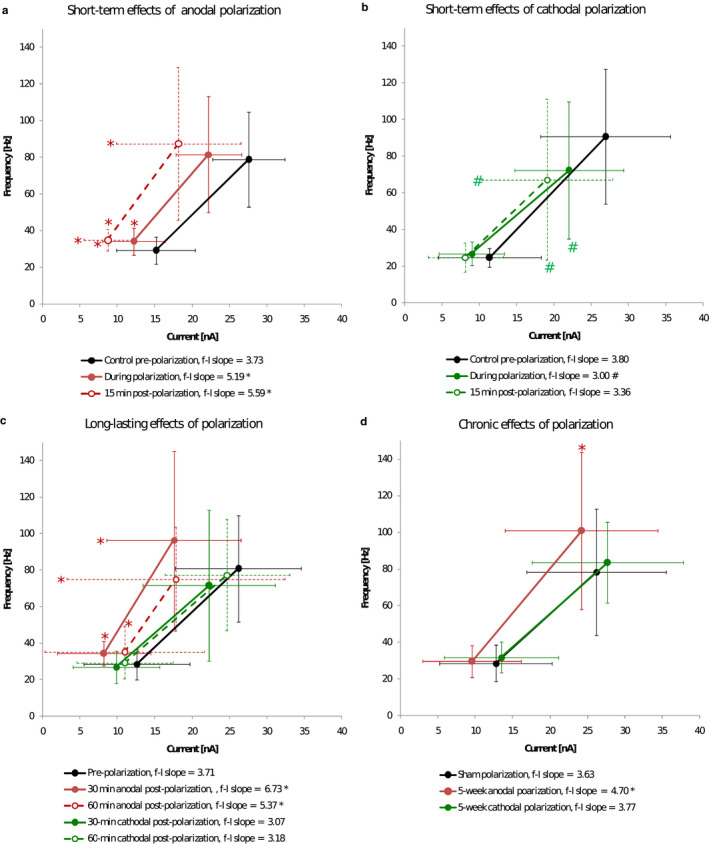
Summary of the changes in the frequency–current (f–I) relationship during rhythmic steady‐state firing (SSF) for MNs subjected to various polarization protocols. The linear relationship between the discharge frequency and injected current was assessed for each MN on the equation *y* = *ax* + *b*, where *a* determines the slope of the relationship in the primary range. Short‐term effects of anodal (a) and cathodal (b) polarization as in Figure 3a and Table 1 (columns 1 and 2) (Bączyk et al., 2019). (c) Long‐lasting effects of polarization as in Figure 3b and Table 1 (columns 3 and 4) (Bączyk et al., 2020a). (d) Chronic effects of polarization as in Figure 3c and Table 1 (column 5) (Bączyk et al., 2020b). Filled and open circles represent the average values for each group, while horizontal and vertical whiskers represent the SD values. “*” indicates significant effect of anodal polarization regarding the minimum and the maximum SSF current, the minimum and the maximum SSF frequency, and the f–I slope, at *p *<* *0.05. “#” indicates significant effect of cathodal polarization for respective parameters, at *p *<* *0.05. (a) and (b) RM ANOVA with a post hoc Tukey's test (for data with normal distribution and equal variance) or the Friedman tests with post hoc analysis of data with paired Kruskal–Wallis or Student's t‐test (for repeated nonparametric comparisons). (c) Two‐way ANOVA with a post hoc Tukey's test. (d) One‐way ANOVA with a post hoc Tukey's test

Both anodal and cathodal tsDCS elicit polarity‐dependent changes in threshold and firing properties of MNs which appear immediately after onset of polarization and outlast the duration of tsDCS application by at least 15 min (Bączyk et al., 2019; Table 1, Figure 4a,b). The major effects of anodal intervention act toward potentiation of MN firing, whereas cathodal polarization acts mainly toward firing inhibition. Moreover, the effects of anodal polarization are generally more pronounced and uniform than those evoked by cathodal polarization.

**TABLE 1:** Summary of relative changes of membrane properties of MNs in response to acute or chronic application of tsDCS in rats

	1	2	3	4	5
	During polarization	15‐min postpolarization period	30‐min postpolarization period	60‐min postpolarization period	5‐week chronic polarization
Anodal polarization					
RMP (mV)	↑ 10%	–	–	–	–
RIN (MΩ)	–	–	–	–	↑ 27%
Rheo (nA)	↓ 23%	↓ 38%	↓ 31%	–	–
VT (mV)	–	↓ 15%	↓ 13%	–	↓ 8%
Cathodal polarization					
RMP (mV)	↓ 12%	–	–	–	–
RIN (MΩ)	–	–	–	–	–
Rheo (nA)	–	↓ 28%	–	–	–
VT (mV)	↓ 20%	–	–	–	–

Columns 1 and 2 indicate short‐term effects (see Figure 3a), based on data recorded from single MNs during, and 15 min after tsDCS (0.1 mA) application, compared to control recordings before the onset of polarization (averaged across MNs in anodal [*N* = 10] or cathodal [*N* = 10] polarization groups, Bączyk et al., 2019); columns 3 and 4 present long‐lasting effects (see Figure 3b), based on data averaged across separate groups of neurons, recorded during the first 30 min (*N* = 22 for anodal tsDCS, *N* = 21, for cathodal tsDCS), and between 30 and 60 min (*N* = 21 for anodal tsDCS, *N* = 22, for cathodal tsDCS) after the offset of tsDCS (0.1 mA), respectively, compared to the prepolarization group (*N* = 36) from which records were made prior to the onset of tsDCS (Bączyk et al., 2020a); column 5 shows chronic effects (see Figure 3c), based on data averaged for MNs recorded after repeated transcutaneous application of anodal (*N* = 39) or cathodal (*N* = 43) tsDCS (0.5 mA, 15 min daily, for 5 weeks), compared to the sham control group (*N* = 41; Bączyk et al., 2020b). Statistically significant changes of respective parameters (an increase or a decrease) are expressed in percentages in regard to prepolarization or sham control values at *p *<* *0.05. Columns 1 and 2, RM ANOVA with a post hoc Tukey's test (for data with normal distribution and equal variance) or the Friedman tests with post hoc analysis of data with paired Kruskal–Wallis or Student's *t*‐test (for repeated nonparametric comparisons). Columns 3 and 4, two‐way ANOVA with a post hoc Tukey's test. Column 5, one‐way ANOVA with a post hoc Tukey's test. RMP, resting membrane potential; *R*_IN_, input resistance; Rheo, rheobase current; VT, voltage threshold for spike generation

Significant long‐lasting effects of anodal tsDCS causing potentiation of firing of MNs were shown to persist in MNs up to 60 min after the offset of polarization (Bączyk et al., 2020a; Table 1, Figure 4c). The effects of cathodal polarization were less prominent and shorter‐lasting, and were not observed 30 min after the offset of tsDCS. These observations are consistent with several other studies in rats and cats reporting that the effects of polarization last up to 2 hr after cessation of the stimuli (Bączyk et al., 2014; Bolzoni & Jankowska, 2015; Bolzoni, Bączyk, et al., 2013; Bolzoni, Pettersson, et al., 2013). The larger impact of anodal tsDCS compared to cathodal tsDCS may appear surprising but marginal effects of cathodal polarization on activity of MNs were also observed in other animal studies (Bolzoni et al., 2013). In vitro experiments combined with computational neuron models (Lafon et al., 2017) suggest that these differences might be explained by the fact that anodal polarization has a synergistic effect on somatic and dendritic compartments, whereas under cathodal polarization the effects on the two compartments tend to cancel each other.

Chronic tsDCS elicits adaptive changes in electrophysiological properties of lumbar spinal MNs due to repeated and consistent alterations in activity of spinal circuitry (Bączyk et al., 2020b). Anodal polarization evokes adaptations in MN properties in such a way that excitability is increased and firing is facilitated, whereas chronic cathodal polarization has no significant effects (Table 1, Figure 4d). Chronic DC polarization can increase the MN excitability and therefore suggests that this technique may be used to deliver neuroprotection in ALS (Bączyk, Alami et al., 2020; Saxena et al., 2013).

**FIGURE 5:**
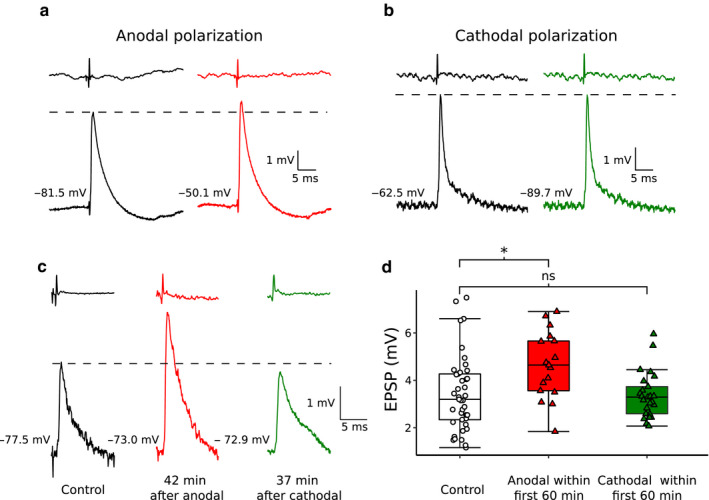
Short‐term and long‐lasting effects of polarization in SOD^G93A^ mice. (a) Monosynaptic EPSPs, evoked by stimulation of the triceps surae nerve, recorded from the same MN, before, and during anodal polarization. (b) as in (a), but records made in a different animal before and during cathodal polarization. (c) Examples of EPSPs recorded in the control (white), long‐lasting anodal (red), and long‐lasting cathodal (green) polarization groups. (d) Distribution of EPSP amplitudes within control, long‐lasting anodal, and long‐lasting cathodal polarization groups. Each data point represents a single MN, while box‐plots cover 25% of the upper and lower data range with horizontal lines showing the median. Notice a strong, 32% increase in EPSP amplitude following anodal polarization (without any change in input resistance). Difference in mean EPSPs amplitude is significant between control and long‐lasting anodal polarization groups (*p* < 0.01, Mann–Whitney test)

The main advantage of DC polarization is that it is not invasive and can be easily used in humans. However preclinical animal studies have to be performed before starting clinical trials. Preliminary results indicate that anodal tsDCS can enhance the excitatory synaptic inputs to MNs in the SOD1^G93A^ mouse model of ALS (Figure 5a,b). Interestingly this effect persists up to 60 min after the end of polarization (Figure 5c,d). Moreover, when ALS mice were chronically treated with daily anodal or cathodal polarization for 2 weeks, EPSP amplitudes of DC‐treated SOD1^G93A^ mice were significantly larger in the anodal polarization group than in the nonpolarized group, whereas no change was seen in the cathodal polarization group (not shown). Altogether these results indicate that in SOD1^G93A^ mice tsDCS evokes polarity‐dependent MN plasticity. Although the functional and survival analysis of tsDCS effects on ALS mice are ongoing, these preliminary findings already provide a proof of concept for further tsDCS application in ALS management.

## 2 CONCLUSION

Recent experiments suggest that vulnerable MNs become intrinsically hypoexcitable (as seen in a loss of their ability to discharge repetitively) and that their excitatory synapses are impaired in the SOD1^G93A^ mice. Pharmacological and chemogenetics interventions that aimed at restoring either the intrinsic excitability or the synaptic strength were shown to ameliorate the disease phenotype. In parallel, other experiments showed that anodal tsDCS enhances the intrinsic excitability of spinal MNs and the effect outlasts the stimulation period. Moreover, preliminary results show that anodal tsDCS also elicits long‐lasting enhancement of EPSPs in SOD1^G93A^ mice, compensating the EPSP impairment observed in these mice. We suggest that chronic anodal tsDCS may be useful in the management of ALS patients.

## References

[phy214706-bib-0001] Ahmed, Z. (2014). Trans‐spinal direct current stimulation modifies spinal cord excitability through synaptic and axonal mechanisms. Physiological Reports, 2(9), e12157. 10.14814/phy2.12157 25263206 10.14814/phy2.12157 PMC4270225

[phy214706-bib-0002] Akamatsu, M. , Yamashita, T. , Hirose, N. , Teramoto, S. , & Kwak, S. (2016). The AMPA receptor antagonist perampanel robustly rescues amyotrophic lateral sclerosis (ALS) pathology in sporadic ALS model mice. Scientific Reports, 6, 28649. 27350567 10.1038/srep28649 PMC4923865

[phy214706-bib-0003] Ardolino, G. , Bocci, T. , Nigro, M. , Vergari, M. , Di Fonzo, A. , Bonato, S. , Cogiamanian, F. , Cortese, F. , Cova, I. , Barbieri, S. , & Priori, A. (2018). Spinal direct current stimulation (tsDCS) in hereditary spastic paraplegias (HSP): A sham‐controlled crossover study. The Journal of Spinal Cord Medicine, 1–8. 10.1080/10790268.2018.1543926 PMC7919872 30508408

[phy214706-bib-0004] Bączyk, M. , Alami, N. O. , Delestrée, N. , Martinot, C. , Tang, L. , Commisso, B. , Bayer, D. , Doisne, N. , Frankel, W. , Manuel, M. , Roselli, F. , & Zytnicki, D. (2020). Synaptic restoration by cAMP/PKA drives activity‐dependent neuroprotection to motoneurons in ALS. Journal of Experimental Medicine, 217(8). https://doi.org/10.1084/jem.20191734. 10.1084/jem.20191734 PMC7398175 32484501

[phy214706-bib-0005] Bączyk, M. , Drzymała‐Celichowska, H. , Mrówczyński, W. , & Krutki, P. (2019). Motoneuron firing properties are modified by trans‐spinal direct current stimulation in rats. Journal of Applied Physiology, 126(5), 1232–1241. 30789288 10.1152/japplphysiol.00803.2018

[phy214706-bib-0006] Bączyk, M. , Drzymała‐Celichowska, H. , Mrówczyński, W. , & Krutki, P. (2020a). Long‐lasting modifications of motoneuron firing properties by trans‐spinal direct current stimulation in rats. European Journal of Neuroscience, 51(8), 1743–1755. 10.1111/ejn.14612 31677210

[phy214706-bib-0007] Bączyk, M. , Drzymała‐Celichowska, H. , Mrówczyński, W. , & Krutki, P. (2020b). Polarity‐dependent adaptations of motoneuron electrophysiological properties after 5‐week transcutaneous spinal direct current stimulation in rats. Journal of Applied Physiology, American Physiological Society. 10.1152/japplphysiol.00301.2020. 32790599

[phy214706-bib-0008] Bączyk, M. , & Jankowska, E. (2014). Presynaptic actions of transcranial and local direct current stimulation in the red nucleus. The Journal of Physiology, 592(19), 4313–4328. 25085891 10.1113/jphysiol.2014.276691 PMC4215779

[phy214706-bib-0009] Bączyk, M. , & Jankowska, E. (2018). Long‐term effects of direct current are reproduced by intermittent depolarization of myelinated nerve fibers. Journal of Neurophysiology, 120(3), 1173–1185. 29924713 10.1152/jn.00236.2018

[phy214706-bib-0010] Bączyk, M. , & Krutki, P. (2020). In vivo intracellular recording of type‐identified rat spinal motoneurons during trans‐spinal direct current stimulation. JoVE (Journal of Visualized Experiments), 159, e61439. 10.3791/61439 32449709

[phy214706-bib-0011] Bączyk, M. , Pettersson, L.‐G. , & Jankowska, E. (2014). Facilitation of ipsilateral actions of corticospinal tract neurons on feline motoneurons by transcranial direct current stimulation. The European Journal of Neuroscience, 40(4), 2628–2640. 24835584 10.1111/ejn.12623 PMC4142254

[phy214706-bib-0012] Bae, Y. C. , Nakamura, T. , Ihn, H. J. , Choi, M. H. , Yoshida, A. , Moritani, M. , Honma, S. , & Shigenaga, Y. (1999). Distribution pattern of inhibitory and excitatory synapses in the dendritic tree of single masseter α‐motoneurons in the cat. The Journal of Comparative Neurology, 414(4), 454–468. 10531539 10.1002/(sici)1096-9861(19991129)414:4<454::aid-cne3>3.0.co;2-7

[phy214706-bib-0013] Bai, X. , Guo, Z. , He, L. , Ren, L. , McClure, M. A. , & Mu, Q. (2019). Different therapeutic effects of transcranial direct current stimulation on upper and lower limb recovery of stroke patients with motor dysfunction: a meta‐analysis. Neural Plasticity, 2019, 1372138. 31827495 10.1155/2019/1372138 PMC6881758

[phy214706-bib-0014] Baldissera, F. , Hultborn, H. , & Illert, M. (1981). Integration in spinal neuronal systems, handbook of physiology, The nervous system. Motor Control,American Physiological Society.

[phy214706-bib-0015] Bellingham, M. C. (2013). Pre‐ and postsynaptic mechanisms underlying inhibition of hypoglossal motor neuron excitability by riluzole. Journal of Neurophysiology, 110, 1047–1061. 23741042 10.1152/jn.00587.2012

[phy214706-bib-0016] Bensimon, G. , Lacomblez, L. , Meininger, V. , & the ALS, Riluzole Study Group (1994). A controlled trial of riluzole in amyotrophic lateral sclerosis. New England Journal of Medicine, 330(9), 585–591. 10.1056/NEJM199403033300901 8302340

[phy214706-bib-0017] Berg, R. W. , Alaburda, A. , & Hounsgaard, J. (2007). Balanced inhibition and excitation drive spike activity in spinal half‐centers. Science, 351(5810), 390–393. 10.1126/science.1134960 17234950

[phy214706-bib-0018] Berra, E. , Bergamaschi, R. , De Icco, R. , Dagna, C. , Perrotta, A. , Rovaris, M. , Grasso, M. G. , Anastasio, M. G. , Pinardi, G. , Martello, F. , Tamburin, S. , Sandrini, G. , & Tassorelli, C. (2019). The effects of transcutaneous spinal direct current stimulation on neuropathic pain in multiple sclerosis: Clinical and neurophysiological assessment. Frontiers in Human Neuroscience, 13, 31. 30809137 10.3389/fnhum.2019.00031 PMC6379270

[phy214706-bib-0019] Berry, H. R. , Tate, R. J. , & Conway, B. A. (2017). Transcutaneous spinal direct current stimulation induces lasting fatigue resistance and enhances explosive vertical jump performance. PLoS One, 12(4), e0173846. 28379980 10.1371/journal.pone.0173846 PMC5381869

[phy214706-bib-0020] Bocci, T. , Marceglia, S. , Vergari, M. , Cognetto, V. , Cogiamanian, F. , Sartucci, F. , & Priori, A. (2015). Transcutaneous spinal direct current stimulation modulates human corticospinal system excitability. Journal of Neurophysiology, 114(1), 440–446. 25925328 10.1152/jn.00490.2014 PMC4509392

[phy214706-bib-0021] Bocci, T. , Vannini, B. , Torzini, A. , Mazzatenta, A. , Vergari, M. , Cogiamanian, F. , Priori, A. , & Sartucci, F. (2014). Cathodal transcutaneous spinal direct current stimulation (tsDCS) improves motor unit recruitment in healthy subjects. Neuroscience Letters, 578, 75–79. 24970753 10.1016/j.neulet.2014.06.037

[phy214706-bib-0022] Bolzoni, F. , Bączyk, M. , & Jankowska, E. (2013). Subcortical effects of transcranial direct current stimulation in the rat. The Journal of Physiology, 591(16), 4027–4042. 23774279 10.1113/jphysiol.2013.257063 PMC3764643

[phy214706-bib-0023] Bolzoni, F. , Esposti, R. , Bruttini, C. , Zenoni, G. , Jankowska, E. , & Cavallari, P. (2017). Direct current stimulation modulates the excitability of the sensory and motor fibres in the human posterior tibial nerve, with a long‐lasting effect on the H‐reflex. European Journal of Neuroscience, 46(9), 2499–2506. 10.1111/ejn.13696 28892581

[phy214706-bib-0024] Bolzoni, F. , & Jankowska, E. (2015). Presynaptic and postsynaptic effects of local cathodal DC polarization within the spinal cord in anaesthetized animal preparations. The Journal of Physiology, 593(4), 947–966. 25416625 10.1113/jphysiol.2014.285940 PMC4398531

[phy214706-bib-0025] Bolzoni, F. , Pettersson, L.‐G. , & Jankowska, E. (2013). Evidence for long‐lasting subcortical facilitation by transcranial direct current stimulation in the cat. The Journal of Physiology, 591(13), 3381–3399. 23507876 10.1113/jphysiol.2012.244764 PMC3717234

[phy214706-bib-0026] Bories, C. , Amendola, J. , Lamotte d’Incamps, B. , & Durand, J. (2007). Early electrophysiological abnormalities in lumbar motoneurons in a transgenic mouse model of amyotrophic lateral sclerosis. The European Journal of Neuroscience, 25(2), 451–459. 17284186 10.1111/j.1460-9568.2007.05306.x

[phy214706-bib-0027] Choi, Y.‐A. , Kim, Y. , & Shin, H.‐I. (2019). Pilot study of feasibility and effect of anodal transcutaneous spinal direct current stimulation on chronic neuropathic pain after spinal cord injury. Spinal Cord, 57(6), 461–470. 30700853 10.1038/s41393-019-0244-x

[phy214706-bib-0028] Cogiamanian, F. , Vergari, M. , Pulecchi, F. , Marceglia, S. , & Priori, A. (2008). Effect of spinal transcutaneous direct current stimulation on somatosensory evoked potentials in humans. Clinical Neurophysiology, 119(11), 2636–2640. 18786856 10.1016/j.clinph.2008.07.249

[phy214706-bib-0029] Cogiamanian, F. , Vergari, M. , Schiaffi, E. , Marceglia, S. , Ardolino, G. , Barbieri, S. , & Priori, A. (2011). Transcutaneous spinal cord direct current stimulation inhibits the lower limb nociceptive flexion reflex in human beings. Pain, 152(2), 370–375. 21159430 10.1016/j.pain.2010.10.041

[phy214706-bib-0030] Coombs, J. S. , Curtis, D. R. , & Eccles, J. C. (1957). The interpretation of spike potentials of motoneurones. The Journal of Physiology, 139(2), 198–231. 13492209 10.1113/jphysiol.1957.sp005887 PMC1358725

[phy214706-bib-0031] De Carvalho, M. , Pinto, S. , Costa, J. , Evangelista, T. , Ohana, B. , & Pinto, A. (2010). A randomized, placebo‐controlled trial of memantine for functional disability in amyotrophic lateral sclerosis. Amyotrophic Lateral Sclerosis, 11(5), 456–460. 20565333 10.3109/17482968.2010.498521

[phy214706-bib-0032] Delestrée, N. , Manuel, M. , Iglesias, C. , Elbasiouny, S. M. , Heckman, C. J. , & Zytnicki, D. (2014). Adult spinal motoneurones are not hyperexcitable in a mouse model of inherited amyotrophic lateral sclerosis. The Journal of Physiology, 592(7), 1687–1703. 24445319 10.1113/jphysiol.2013.265843 PMC3979619

[phy214706-bib-0033] Devlin, A.‐C. , Burr, K. , Borooah, S. , Foster, J. D. , Cleary, E. M. , Geti, I. , Vallier, L. , Shaw, C. E. , Chandran, S. , & Miles, G. B. (2015). Human iPSC‐derived motoneurons harbouring TARDBP or C9ORF72 ALS mutations are dysfunctional despite maintaining viability. Nature Communications, 6, 5999. 10.1038/ncomms6999 PMC4338554 25580746

[phy214706-bib-0034] Eccles, J. C. , Kostyuk, P. G. , & Schmidt, R. F. (1962). The effect of electric polarization of the spinal cord on central afferent fibres and on their excitatory synaptic action. The Journal of Physiology, 162(1), 138–150. 13889056 10.1113/jphysiol.1962.sp006920 PMC1359645

[phy214706-bib-0035] Fang, T. , Al Khleifat, A. , Meurgey, J.‐H. , Jones, A. , Leigh, P. N. , Bensimon, G. , & Al‐Chalabi, A. (2018). Stage at which riluzole treatment prolongs survival inpatients with amyotrophic lateral sclerosis: a retrospective analysis of data from a dose‐ranging study. Lancet Neurology, 17, 416–422. 29525492 10.1016/S1474-4422(18)30054-1 PMC5899963

[phy214706-bib-0036] Hegedus, J. , Putman, C. T. , Tyreman, N. , & Gordon, T. (2008). Preferential motor unit loss in the SOD1 G93A transgenic mouse model of amyotrophic lateral sclerosis. The Journal of Physiology, 586(14), 3337–3351. 18467368 10.1113/jphysiol.2007.149286 PMC2538809

[phy214706-bib-0037] Heide, A. C. , Winkler, T. , Helms, H. J. , Nitsche, M. A. , Trenkwalder, C. , Paulus, W. , & Bachmann, C. G. (2014). Effects of transcutaneous spinal direct current stimulation in idiopathic restless legs patients. Brain Stimulation, 7(5), 636–642. 25216650 10.1016/j.brs.2014.06.008

[phy214706-bib-0038] Hodgkin, A. L. , & Huxley, A. F. (1952). A quantitative description of membrane current and its application to conduction and excitation in nerve. The Journal of Physiology, 117(4), 500–544. 12991237 10.1113/jphysiol.1952.sp004764 PMC1392413

[phy214706-bib-0039] Hubli, M. , Dietz, V. , Schrafl‐Altermatt, M. , & Bolliger, M. (2013). Modulation of spinal neuronal excitability by spinal direct currents and locomotion after spinal cord injury. Clinical Neurophysiology, 124(6), 1187–1195. 23415451 10.1016/j.clinph.2012.11.021

[phy214706-bib-0040] Ilieva, H. , Polymenidou, M. , & Cleveland, D. W. (2009). Non–cell autonomous toxicity in neurodegenerative disorders: ALS and beyond. The Journal of Cell Biology, 187(6), 761–772. 19951898 10.1083/jcb.200908164 PMC2806318

[phy214706-bib-0041] Jankowska, E. (2017). Spinal control of motor outputs by intrinsic and externally induced electric field potentials. Journal of Neurophysiology, 118(2), 1221–1234. 28539396 10.1152/jn.00169.2017 PMC5547263

[phy214706-bib-0042] Jankowska, E. , Kaczmarek, D. , Bolzoni, F. , & Hammar, I. (2016). Evidence that some long‐lasting effects of direct current in the rat spinal cord are activity‐independent. The European Journal of Neuroscience, 43(10), 1400–1411. 26990901 10.1111/ejn.13238

[phy214706-bib-0043] Jensen, D. , Kadlecova, M. , Allodi, I. , & Meehan, F. (2020). Spinal motoneurones are intrinsically more responsive in the adult G93A SOD1 mouse model of amyotrophic lateral sclerosis. The Journal of Physiology. 10.1113/JP280097. Online ahead of print. 32716521

[phy214706-bib-0044] Joo, I. S. , Hwang, D. H. , Seok, J. I. , Shin, S. K. , & Kim, S. U. (2007). Oral administration of memantine prolongs survival in a transgenic mouse model of amyotrophic lateral sclerosis. Journal of Clinical Neurology, 3, 181–186. 19513129 10.3988/jcn.2007.3.4.181 PMC2686946

[phy214706-bib-0045] Kaczmarek, D. , & Jankowska, E. (2018). DC‐evoked modulation of excitability of myelinated nerve fibers and their terminal branches. Differences in Sustained Effects of DC. Neuroscience, 374, 236–249. 29421432 10.1016/j.neuroscience.2018.01.036

[phy214706-bib-0046] Kaczmarek, D. , Ristikankare, J. , & Jankowska, E. (2017). Does trans‐spinal and local DC polarization affect presynaptic inhibition and post‐activation depression? The Journal of Physiology, 595(5), 1743–1761. 27891626 10.1113/JP272902 PMC5330874

[phy214706-bib-0047] Kanning, K. C. , Kaplan, A. , & Henderson, C. E. (2010). Motor neuron diversity in development and disease. Annual Review of Neuroscience, 33, 409–440. 10.1146/annurev.neuro.051508.135722 20367447

[phy214706-bib-0048] Knikou, M. , Dixon, L. , Santora, D. , & Ibrahim, M. M. (2015). Transspinal constant‐current long‐lasting stimulation: a new method to induce cortical and corticospinal plasticity. Journal of Neurophysiology, 114(3), 1486–1499. 26108955 10.1152/jn.00449.2015 PMC4556848

[phy214706-bib-0049] Kuck, A. , Stegeman, D. F. , van der Kooij, H. , & van Asseldonk, E. H. F. (2018). Changes in H‐reflex recruitment after trans‐spinal direct current stimulation with multiple electrode configurations. Frontiers in Neuroscience, 12, 151. 29643759 10.3389/fnins.2018.00151 PMC5882846

[phy214706-bib-0050] Kuo, J. J. , Lee, R. H. , Zhang, L. , & Heckman, C. J. (2006). Essential role of the persistent sodium current in spike initiation during slowly rising inputs in mouse spinal neurones. Journal of Physiology, 574(3), 819–834. 10.1113/jphysiol.2006.107094 PMC1817738 16728453

[phy214706-bib-0051] Kuo, M.‐F. , Chen, P.‐S. , & Nitsche, M. A. (2017). The application of tDCS for the treatment of psychiatric diseases. International Review of Psychiatry (Abingdon, England), 29(2), 146–167. 10.1080/09540261.2017.1286299 28523976

[phy214706-bib-0052] Lafon, B. , Rahman, A. , Bikson, M. , & Parra, L. C. (2017). Direct current stimulation alters neuronal input/output function. Brain Stimulation, 10(1), 36–45. 27717601 10.1016/j.brs.2016.08.014 PMC5774009

[phy214706-bib-0053] Lalancette‐Hebert, M. , Sharma, A. , Lyashchenko, A. K. , & Shneider, N. A. (2016). Gamma motor neurons survive and exacerbate alpha motor neuron degeneration in ALS. Proceedings of National Academy of Sciences of the United States of America, 113, 8316–8325. 10.1073/pnas.1605210113 PMC5187676 27930290

[phy214706-bib-0054] Lamanauskas, N. , & Nistri, A. (2008). Riluzole blocks persistent Na+ and Ca2+ currents and modulates release of glutamate via presynaptic NMDA receptors on neonatal rat hypoglossal motoneurons in vitro. European Journal of Neuroscience, 27, 2501–2514. 10.1111/j.1460-9568.2008.06211.x 18445055

[phy214706-bib-0055] Lamy, J.‐C. , Ho, C. , Badel, A. , Arrigo, R. T. , & Boakye, M. (2012). Modulation of soleus H reflex by spinal DC stimulation in humans. Journal of Neurophysiology, American Physiological Society, 108(3), 906–914. 22623482 10.1152/jn.10898.2011

[phy214706-bib-0056] Lenoir, C. , Jankovski, A. , & Mouraux, A. (2018). Anodal transcutaneous spinal direct current stimulation (tsDCS) selectively inhibits the synaptic efficacy of nociceptive transmission at spinal cord level. Neuroscience, 393, 150–163. 30321585 10.1016/j.neuroscience.2018.10.007 PMC6364802

[phy214706-bib-0057] Leroy, F. , Lamotte d’Incamps, B. , Imhoff‐Manuel, R. D. , & Zytnicki, D. (2014). Early intrinsic hyperexcitability does not contribute to motoneuron degeneration in amyotrophic lateral sclerosis. ELife, 3, e04046. 25313866 10.7554/eLife.04046 PMC4227046

[phy214706-bib-0058] Li, Y. , Hari, K. , Lucas‐Osma, A. M. , Fenrich, K. K. , Bennett, D. J. , Hammar, I. , & Jankowska, E. (2020). Branching points of primary afferent fibers are vital for the modulation of fiber excitability by epidural DC polarization and by GABA in the rat spinal cord. Journal of Neurophysiology, American Physiological Society, 124(1), 49–62. 10.1152/jn.00161.2020 PMC7474458 32459560

[phy214706-bib-0059] Magnus, C. J. , Lee, P. H. , Atasoy, D. , Su, H. H. , Looger, L. L. , & Sternson, S. M. (2011). Chemical and genetic engineering of selective ion channel‐ligand interactions. Science 333(6047):1292–1296. 21885782 10.1126/science.1206606 PMC3210548

[phy214706-bib-0060] Manuel, M. (2020). Sub‐optimal discontinuous current‐clamp switching rates lead to deceptive mouse neuronal firing. bioRxiv, 10.1101/2020.08.13.250134. PMC7901151 33446514

[phy214706-bib-0061] Martin, E. , Cazenave, W. , Cattaert, D. , & Branchereau, P. (2013). Embryonic alteration of motoneuronal morphology induces hyperexcitability in the mouse model of amyotrophic lateral sclerosis. Neurobiology of Disease, 54, 116–126. 23466698 10.1016/j.nbd.2013.02.011

[phy214706-bib-0062] Martínez‐Silva, M. D. L. , Imhoff‐Manuel, R. D. , Sharma, A. , Heckman, C. J. , Shneider, N. A. , Roselli, F. , Zytnicki, D. , & Manuel, M. (2018). Hypoexcitability precedes denervation in the large fast‐contracting motor units in two unrelated mouse models of ALS. Elife, 7, 10.7554/eLife.30955. PMC5922970 29580378

[phy214706-bib-0063] Meehan, C. F. , Moldovan, M. , Marklund, S. L. , Graffmo, K. S. , Nielsen, J. B. , & Hultborn, H. (2010). Intrinsic properties of lumbar motor neurones in the adult G127insTGGG superoxide dismutase‐1 mutant mouse in vivo: Evidence for increased persistent inward currents. Acta Physiologica, 200, 361–376. 20874803 10.1111/j.1748-1716.2010.02188.x

[phy214706-bib-0064] Miller, R. G. , Mitchell, J. D. , Lyon, M. , & Moore, D. H. (2003). Riluzole for amyotrophic lateral sclerosis (ALS)/motor neuron disease (MND). Amyotrophic Lateral Sclerosis and Other Motor Neuron Disorders, 4(3), 191–206. 13129806

[phy214706-bib-0065] Murray, L. M. , Tahayori, B. , & Knikou, M. (2018). Transspinal direct current stimulation produces persistent plasticity in human motor pathways. Scientific Reports, 8(1), 717. 29335430 10.1038/s41598-017-18872-z PMC5768745

[phy214706-bib-0066] Naujock, M. , Stanslowsky, N. , Bufler, S. , Naumann, M. , Reinhardt, P. , Sterneckert, J. , Kefalakes, E. , Kassebaum, C. , Bursch, F. , Lojewski, X. , Storch, A. , Frickenhaus, M. , Boeckers, T. M. , Putz, S. , Demestre, M. , Liebau, S. , Klingenstein, M. , Ludolph, A. C. , Dengler, R. , … Petri, S. (2016). 4‐Aminopyridine induced activity rescues hypoexcitable motor neurons from amyotrophic lateral sclerosis patient‐derived induced pluripotent stem cells. Stem Cells (Dayton, Ohio), 34(6), 1563–1575. 10.1002/stem.2354 26946488

[phy214706-bib-0067] Nelson, P. G. (1966). Interaction between spinal motoneurons of the cat. Journal of Neurophysiology, 29(2), 275–287. 5927462 10.1152/jn.1966.29.2.275

[phy214706-bib-0068] Netzahualcoyotzi, C. , & Tapia, R. (2015). Degeneration of spinal motor neurons by chronic AMPA‐induced excitotoxicity in vivo and protection by energy substrates. Acta Neuropathologica Communications, 3, 27. 25968178 10.1186/s40478-015-0205-3 PMC4429664

[phy214706-bib-0069] Nitsche, M. A. , & Paulus, W. (2000). Excitability changes induced in the human motor cortex by weak transcranial direct current stimulation. The Journal of Physiology, 527(Pt 3), 633–639. 10990547 10.1111/j.1469-7793.2000.t01-1-00633.x PMC2270099

[phy214706-bib-0070] Noga, B. R. , Fortier, P. A. , Kriellaars, D. J. , Dai, X. , Detillieux, G. R. , & Jordan, L. M. (1995). Field potential mapping of neurons in the lumbar spinal cord activated following stimulation of the mesencephalic locomotor region. The Journal of Neuroscience, 15(3 Pt 2), 2203–2217. 7891162 10.1523/JNEUROSCI.15-03-02203.1995 PMC6578129

[phy214706-bib-0071] Paget‐Blanc, A. , Chang, J. L. , Saul, M. , Lin, R. , Ahmed, Z. , & Volpe, B. T. (2019). Non‐invasive treatment of patients with upper extremity spasticity following stroke using paired trans‐spinal and peripheral direct current stimulation. Bioelectronic Medicine, 5 10.1186/s42234-019-0028-9. PMC7098221 32232101

[phy214706-bib-0072] Pambo‐Pambo, A. , Durand, J. , & Gueritaud, J.‐P. (2009). Early excitability changes in lumbar motoneurons of transgenic SOD1G85R and SOD1G(93A‐Low) mice. Journal of Neurophysiology, 102(6), 3627–3642. 19828728 10.1152/jn.00482.2009

[phy214706-bib-0073] Pascuzzi, R. M. , Shefner, J. , Chappell, A. S. , Bjerke, J. S. , Tamura, R. , Chaudhry, V. , Clawson, L. , Haas, L. , & Rothstein, J. D. (2010). A phase II trial of talampanel in subjects with amyotrophic lateral sclerosis. Amyotrophic Lateral Sclerosis, 11(3), 266–271. 19961264 10.3109/17482960903307805

[phy214706-bib-0074] Pieri, M. , Albo, F. , Gaetti, C. , Spalloni, A. , Bengtson, C. P. , Longone, P. , Cavalcanti, S. , & Zona, C. (2003). Altered excitability of motor neurons in a transgenic mouse model of familial amyotrophic lateral sclerosis. Neuroscience Letters, 351(3), 153–156. 14623129 10.1016/j.neulet.2003.07.010

[phy214706-bib-0075] Pun, S. , Santos, A. F. , Saxena, S. , Xu, L. , & Caroni, P. (2006). Selective vulnerability and pruning of phasic motoneuron axons in motoneuron disease alleviated by CNTF. Nature Neuroscience, 9(3), 408–419. 16474388 10.1038/nn1653

[phy214706-bib-0076] Quinlan, K. A. , Schuster, J. E. , Fu, R. , Siddique, T. , & Heckman, C. J. (2011). Altered postnatal maturation of electrical properties in spinal motoneurons in a mouse model of amyotrophic lateral sclerosis. The Journal of Physiology, 589(Pt 9), 2245–2260. 21486770 10.1113/jphysiol.2010.200659 PMC3098701

[phy214706-bib-0077] Ramger, B. C. , Bader, K. A. , Davies, S. P. , Stewart, D. A. , Ledbetter, L. S. , Simon, C. B. , & Feld, J. A. (2019). Effects of non‐invasive brain stimulation on clinical pain intensity and experimental pain sensitivity among individuals with central post‐stroke pain: A systematic review. Journal of Pain Research, 12, 3319–3329. 31853195 10.2147/JPR.S216081 PMC6916700

[phy214706-bib-0078] Sareen, D. , O'Rourke, J. G. , Meera, P. , Muhammad, A. K. M. G. , Grant, S. , Simpkinson, M. , Bell, S. , Carmona, S. , Ornelas, L. , Sahabian, A. , Gendron, T. , Petrucelli, L. , Baughn, M. , Ravits, J. , Harms, M. B. , Rigo, F. , Bennett, C. F. , Otis, T. S. , Svendsen, C. N. , & Baloh, R. H. (2013). Targeting RNA foci in iPSC‐derived motor neurons from ALS patients with a C9ORF72 repeat expansion. Science Translational Medicine, 5(208), 208ra149. 10.1126/scitranslmed.3007529 PMC4090945 24154603

[phy214706-bib-0079] Saxena, S. , Roselli, F. , Singh, K. , Leptien, K. , Julien, J.‐P. , Gros‐Louis, F. , & Caroni, P. (2013). Neuroprotection through excitability and mTOR required in ALS motoneurons to delay disease and extend survival. Neuron, 80(1), 80–96. 24094105 10.1016/j.neuron.2013.07.027

[phy214706-bib-0080] Sharma, A. , Lyashchenko, A. K. , Lu, L. , Nasrabady, S. E. , Elmaleh, M. , Mendelsohn, M. , Nemes, A. , Tapia, J. C. , Mentis, G. Z. , & Shneider, N. A. (2016). ALS‐associated mutant FUS induces selective motor neuron degeneration through toxic gain of function. Nature Communications, 7(1), 10465. 10.1038/ncomms10465 PMC4742863 26842965

[phy214706-bib-0081] Truini, A. , Vergari, M. , Biasiotta, A. , La Cesa, S. , Gabriele, M. , Di Stefano, G. , Cambieri, C. , Cruccu, G. , Inghilleri, M. , & Priori, A. (2011). Transcutaneous spinal direct current stimulation inhibits nociceptive spinal pathway conduction and increases pain tolerance in humans. European Journal of Pain (London, England), 15(10), 1023–1027. 10.1016/j.ejpain.2011.04.009 21576030

[phy214706-bib-0082] Van Damme, P. , Leyssen, M. , Callewaert, G. , Robberecht, W. , Van, D. B. L. (2003). The AMPA receptor antagonist NBQX prolongs survival in a transgenic mouse model of amyotrophic lateral sclerosis. Neuroscience Letters, 343, 81–84. 12759169 10.1016/s0304-3940(03)00314-8

[phy214706-bib-0083] Van Den Bosch, L. , Van Damme, P. , Bogaert, E. , & Robberecht, W. (2006). The role of excitotoxicity in the pathogenesis of amyotrophic lateral sclerosis. Biochimica et Biophysica Acta, 1762(11–12), 1068–1082. 16806844 10.1016/j.bbadis.2006.05.002

[phy214706-bib-0084] van Zundert, B. , Peuscher, M. H. , Hynynen, M. , Chen, A. , Neve, R. L. , Brown, R. H. , Constantine‐Paton, M. , & Bellingham, M. C. (2008). Neonatal neuronal circuitry shows hyperexcitable disturbance in a mouse model of the adult‐onset neurodegenerative disease amyotrophic lateral sclerosis. The Journal of Neuroscience, 28(43), 10864–10874. 18945894 10.1523/JNEUROSCI.1340-08.2008 PMC3844745

[phy214706-bib-0085] Venugopal, S. , Hsiao, C.‐F. , Sonoda, T. , Wiedau‐Pazos, M. , & Chandler, S. H. (2015). Homeostatic dysregulation in membrane properties of masticatory motoneurons compared with oculomotor neurons in a mouse model for amyotrophic lateral sclerosis. The Journal of Neuroscience, 35(2), 707–720. 25589764 10.1523/JNEUROSCI.1682-14.2015 PMC4293417

[phy214706-bib-0086] Wainger, B. J. , Kiskinis, E. , Mellin, C. , Wiskow, O. , Han, S. S. W. , Sandoe, J. , Perez, N. P. , Williams, L. A. , Lee, S. , Boulting, G. , Berry, J. D. , Brown, R. H. , Cudkowicz, M. E. , Bean, B. P. , Eggan, K. , & Woolf, C. J. (2014). Intrinsic membrane hyperexcitability of amyotrophic lateral sclerosis patient‐derived motor neurons. Cell Reports, 7(1), 1–11. 24703839 10.1016/j.celrep.2014.03.019 PMC4023477

[phy214706-bib-0087] Weiss, M. D. , Macklin, E. A. , Simmons, Z. , Knox, A. S. , Greenblatt, D. J. , Atassi, N. , Graves, M. , Parziale, N. , Salameh, J. S. , Quinn, C. , Brown, R. H. Jr , Distad, J. B. , Trivedi, J. , Shefner, J. M. , Barohn, R. J. , Pestronk, A. , Swenson, A. , & Cudkowicz, M. E. ; Mexiletine ALS Study Group . (2016). A randomized trial of mexiletine in ALS: Safety and effects on muscle cramps and progression. Neurology, 86(16), 1474–1481. 26911633 10.1212/WNL.0000000000002507 PMC4836879

[phy214706-bib-0088] Winkler, T. , Hering, P. , & Straube, A. (2010). Spinal DC stimulation in humans modulates post‐activation depression of the H‐reflex depending on current polarity. Clinical Neurophysiology, 121(6), 957–961. 20153248 10.1016/j.clinph.2010.01.014

[phy214706-bib-0089] Wobst, H. J. , Mack, K. L. , Brown, D. G. , Brandon, N. J. , & Shorter, J. (2020). The clinical trial landscape in amyotrophic lateral sclerosis‐Past, present, and future. Medicinal Research Reviews, 40(4), 1352–1384. 32043626 10.1002/med.21661 PMC7417284

[phy214706-bib-0090] Zhao, C. , Devlin, A.‐C. , Chouhan, A. K. , Selvaraj, B. T. , Stavrou, M. , Burr, K. , Brivio, V. , He, X. , Mehta, A. R. , Story, D. , Shaw, C. E. , Dando, O. , Hardingham, G. E. , Miles, G. B. , & Chandran, S. (2020). Mutant C9orf72 human iPSC‐derived astrocytes cause non‐cell autonomous motor neuron pathophysiology. Glia, 68(5), 1046–1064. 31841614 10.1002/glia.23761 PMC7078830

